# Applications of Nanovaccines for Disease Prevention in Cattle

**DOI:** 10.3389/fbioe.2020.608050

**Published:** 2020-12-11

**Authors:** Teresia W. Maina, Elizabeth A. Grego, Paola M. Boggiatto, Randy E. Sacco, Balaji Narasimhan, Jodi L. McGill

**Affiliations:** ^1^Department of Veterinary Microbiology and Preventive Medicine, Iowa State University, Ames, IA, United States; ^2^Department of Chemical and Biological Engineering, Iowa State University, Ames, IA, United States; ^3^Infectious Bacterial Diseases Research Unit, National Animal Disease Center, Agricultural Research Service, United States Department of Agriculture, Ames, IA, United States; ^4^Ruminant Diseases and Immunology Research Unit, National Animal Disease Center, Agricultural Research Service, United States Department of Agriculture, Ames, IA, United States

**Keywords:** nanovaccine, infectious diseases, cattle, immune response, nanocarriers

## Abstract

Vaccines are one of the most important tools available to prevent and reduce the incidence of infectious diseases in cattle. Despite their availability and widespread use to combat many important pathogens impacting cattle, several of these products demonstrate variable efficacy and safety in the field, require multiple doses, or are unstable under field conditions. Recently, nanoparticle-based vaccine platforms (nanovaccines) have emerged as promising alternatives to more traditional vaccine platforms. In particular, polymer-based nanovaccines provide sustained release of antigen payloads, stabilize such payloads, and induce enhanced antibod- and cell-mediated immune responses, both systemically and locally. To improve vaccine administrative strategies and efficacy, they can be formulated to contain multiple antigenic payloads and have the ability to protect fragile proteins from degradation. Nanovaccines are also stable at room temperature, minimizing the need for cold chain storage. Nanoparticle platforms can be synthesized for targeted delivery through intranasal, aerosol, or oral administration to induce desired mucosal immunity. In recent years, several nanovaccine platforms have emerged, based on biodegradable and biocompatible polymers, liposomes, and virus-like particles. While most nanovaccine candidates have not yet advanced beyond testing in rodent models, a growing number have shown promise for use against cattle infectious diseases. This review will highlight recent advancements in polymeric nanovaccine development and the mechanisms by which nanovaccines may interact with the bovine immune system. We will also discuss the positive implications of nanovaccines use for combating several important viral and bacterial disease syndromes and consider important future directions for nanovaccine development in beef and dairy cattle.

## Introduction

Infectious diseases in cattle are caused by different classes of pathogens, including viruses, bacteria, fungi, and parasites. Some of the economically important diseases in the cattle industry world-wide include Foot-and-mouth disease (FMD), brucellosis, Johne's disease, anaplasmosis and bovine respiratory disease complex (BRDC), a multifactorial disease involving both viral and bacterial pathogens. Although the clinical signs and symptoms vary depending on the infectious pathogen, most of these diseases have the potential to lead to high morbidity and/or mortality, reduced fertility, and reduced production efficiency, resulting in major economic losses. For example, the costs attributed to anaplasmosis have been estimated to exceed $300 million annually in the United States due to reduced performance, abortions, bull infertility and cow deaths (Zabel and Agusto, 2018). The impact of Johne's disease (caused by infection with *Mycobacterium avium* subspecies *paratuberculosis*) has been estimated to reach as high as $200–250 million annually (Ott et al., 1999; Raizman et al., 2009), while the costs attributed to BRDC reach as high as $3 billion annually worldwide from treatment costs, deaths, and impacts on performance (Watts and Sweeney, 2010). Hence, effective control of infectious diseases is crucial for global animal health and food security.

Infectious diseases are challenging to eradicate, with Rinderpest being the only animal disease formally declared as globally eradicated in 2011 (https://www.oie.int/for-the-media/rinderpest/). The current treatment and control strategies for most infectious diseases include the use of antimicrobials, improved management practices, and vaccinations. Vaccination continues to be an efficient and cost-effective tool for control, and the paucity of effective veterinary vaccines is problematic, compounded by numerous reports of increased antimicrobial resistance and considerable financial burden to the producer from antibiotics use. Given the impact of vaccination on animal health, there is a need to develop new vaccines and improve the efficacy of existing vaccines against these infections.

The use of nanoparticle-based vaccines (i.e., nanovaccines) is emerging as a promising novel approach for vaccination because of competitive advantages over the use of traditional subunit vaccines. Nanovaccines can induce both rapid and long-lasting cellular and humoral immunity (Sekhon and Saluja, 2011; Wagner et al., 2019; Bhardwaj et al., 2020). Nanovaccines are also easily administered through multiple delivery routes, including intranasal, intravenous, transdermal, oral, and can be functionalized to cross the blood brain barrier (McGill et al., 2018; Chenthamara et al., 2019). These vaccine formulations exhibit extended thermal stability at room temperature and do not require a cold chain, unlike traditional subunit vaccines (Wagner-Muñiz et al., 2018). The storage stability of nanovaccines significantly reduces medical storage costs and allows for global delivery to remote locations (Lee et al., 2017). In addition to thermal stability, nanoparticles are capable of maintaining antigen protein stability and functionality for extended periods of time, which is desired to elicit an appropriate immune response (Petersen et al., 2010, 2012; Haughney et al., 2013; Ross et al., 2014; Vela Ramirez et al., 2014; González-Aramundiz et al., 2018; McGill et al., 2018; Liu et al., 2020).

Nanovaccines can be designed to mimic the size and shape of pathogens to promote easy uptake by immune cells (Narasimhan et al., 2016). In addition, they can be linker functionalized with molecules such as alpha-1,2-linked dimannopyranoside, to make them “pathogen-like” for efficient uptake. Once internalized by immune cells, the particles degrade to release encapsulated antigen over a prolonged period of time. Particles are typically synthesized from a multitude of natural and synthetic biocompatible polymeric materials, allowing for a “plug and play” platform. The diversity of materials used to synthesize nanoparticles allows for the manipulation of the particle shape, size, surface charge, and hydrophobicity, and ultimately self-adjuvanting effects. Given these advantages, nanovaccines may be useful for improving vaccine efficacy in agriculture species. In this review, we consider how the use of polymeric nanoparticles as antigen carriers and/or adjuvants may be used to improve vaccine efficacy and stability against common infectious diseases in cattle. We also highlight some distinctive aspects of the bovine immune system, which should be considered when designing and optimizing efficacious nanovaccine formulations.

## Unique Aspects of the Bovine Immune System

The immune system is relatively conserved among mammalian species. Thus, cattle share many aspects of innate and adaptive immune function with humans, rodents, and other animals. However, particular aspects of the bovine immune system are unique and should be considered in the context of infectious diseases and vaccine development. Here, while not meant to be an exhaustive description, we have highlighted a few important components of the immune system in cattle that may be considered for vaccine development and evaluating the efficacy of nanovaccine platforms.

### The Bovine Innate Immune System

#### Pathogen Recognition Receptors

Activation of the innate immune system is based on its ability to recognize so-called alarm (or danger) signals. These signals can come in two forms, exogenous, that is recognition of molecules derived from microorganisms, and endogenous, recognition of dead and dying cells. Exogenous signals are collectively referred to as pathogen-associated molecular patterns (PAMPs), and endogenous signals are referred to as damage-associated molecular patterns (DAMPs). Pattern recognition receptors (PRRs) expressed by various cells recognize PAMPs and DAMPs, and subsequently trigger the activation of the innate immune system.

Toll-like receptors (TLR) are an important family of PRRs that are expressed both on the cell surface and within intracellular compartments of cells. Cattle, similar to humans, express TLR-1 to TLR-10. In general, ligand recognition among mammalian TLR is similar; however, species-specific differences in ligand recognition and subsequent signaling have been identified [reviewed in Werling et al. (2009)]. These species-specific differences may be related to variable regions on the TLR molecule (Hajjar et al., 2002) or may be due to association with co-receptors (Akashi et al., 2001). In other cases, even within a species, differences can be found in the extent of activation depending on the source of the ligand. For example, bovine TLR-4 responds strongly to lipopolysaccharide (LPS) from *Rhodobacter sphaeroides* but weakly to monophosphoryl-lipid A from *Salmonella Minnesota* (Lizundia et al., 2008). Various studies have reported polymorphisms in all 11 bovine TLR across cattle breeds (Cargill and Womack, 2007; Marco et al., 2009; Werling et al., 2009; Seabury et al., 2010; Fisher et al., 2011; Russell et al., 2012). These mutations could result in changes to ligand recognition and may also be associated with disease susceptibility (Fisher et al., 2011; Russell et al., 2012). While TLR-ligand pairs are generally conserved across species, it is important to note these differences, especially as we often extrapolate findings from one species to another. This is particularly important in the context new adjuvants for vaccination strategies, which often target TLRs.

C-type lectins are receptors that recognize carbohydrates, and some C-type lectins specifically recognize carbohydrates on the surface of microorganisms and are therefore termed PRRs. The main C-type lectins involved in pathogen recognition include dectin-1, dectin-2, DEC205, and the mannose receptor. Dectin-1 is expressed by bovine macrophages, monocytes and dendritic cells, while dectin-2 is expressed by bovine Langerhans cells in the skin. Various bovine DC subsets have been shown to express DEC205. In addition to their potential role adjuvant targets (Decout et al., 2017), C-type lectins can be exploited to target antigens or therapeutics to specific cell subtypes (Chiffoleau, 2018).

Retinoic acid-inducible gene I (RIG-I) and other closely related RIG-I like receptors (RLR) are cytosolic PRR that recognize uncapped 5′-triphosphate RNA or double-stranded RNA. RLR are widely expressed by a variety of cell and tissue types, where they contribute to the innate immune response against several RNA viruses via the induction of the Type I IFN response and early inflammatory responses. A single polymorphism has been reported in bovine RIG-I (Cargill et al., 2006), although it is unknown if this single nucleotide polymorphism is associated with any increased or decreased susceptibility to disease. In cattle, RLR are important for innate recognition of BVDV, FMDV and bovine herpesvirus-4 (Carneiro et al., 2017; Li et al., 2018). RLR have shown some promise as potential adjuvant targets in cattle (El-Attar et al., 2015).

#### Cells of the Innate Immune system

Innate immune cells in cattle are similar to those described in other species. Bovine NK cells can be identified using NKp46 (CD335) and can generally be classified as NKp46^+^ CD3^−^ CD2^−^ CD25^+^ CD8^+^ cells (Storset et al., 2004). Similar to mouse and human studies, cattle NK cells have been identified in multiple tissues including spleen, lung, liver, and various lymph nodes (Storset et al., 2004; Goff et al., 2006; Boysen et al., 2008).

Peripheral blood monocytes comprise a heterogenous population of white blood cells with distinct surface markers and functional phenotypes. In cattle, CD172a serves as a panmarker for monocytes (Hussen et al., 2013), which is in contrast to that observed for human and mice monocytes where CD115 is used to identify monocyte populations (Auffray et al., 2009). As with most species, expression of CD14 (the LPS receptor) and CD16 (Fcγ receptor III) can be used to identify monocyte subsets. Classical monocytes (cM) defined as CD14^++^ CD16^−^, intermediate monocytes (intM) as CD14^++^ CD16^+^, and non-classical monocytes (ncM) as CD14^−^ CD16^++^ (Hussen et al., 2013). Functional heterogeneity can also be defined in bovine monocytes; cM have the highest phagocytic ability while intM tend to have the strongest production of reactive oxygen species and pro-inflammatory cytokines. Therefore, in general, cM and intM are thought to mediate inflammatory processes. This differs from human monocytes, where intM and ncM are described as the pro-inflammatory subsets (Ziegler-Heitbrock, 2015).

Polymorphonuclear (PMN) leukocytes, or neutrophils, play a major role in innate immunity as the first line of cellular defense against pathogens. As with other species, bovine neutrophils have a short life span of a few days, and are constantly replaced. In cattle, neutrophils make up about 20–30% of total blood leukocytes, but the majority are sequestered in capillaries in the liver, spleen, lung, and bone marrow. Bovine neutrophils express a variety of receptors on their surface that facilitate many functions. Similar to bovine monocytes and macrophages, bovine neutrophils express CD14 (Paape et al., 1996), which recognizes LPS bound to LPS binding protein (LPB) (Wright et al., 1990). Opsonin receptors include complement receptor 1 (CR1) and Fc receptors (FcR). IgM and IgG_2_ are the main opsonizing antibodies in cattle (Anderson et al., 1986; Guidry et al., 1993) and unlike human neutrophils, which primarily express FcγRI and FcγRII, bovine neutrophils express a special receptor for IgG2, the Fcγ_2_ receptor (Zhang et al., 1995). Adhesion receptors are also expressed by bovine neutrophils including, CR3 (CD11b/CD18) and CD62L. These molecules mediate adhesion and rolling onto endothelial surfaces, respectively.

The primary function of dendritic cells (DC) is to serve as antigen presenting cells (APC) to T and B cells. Although not as well-characterized as in humans and mice, DC in cattle are composed of a heterogenous population of cells. They are divided into three main groups, including bone marrow derived (BMDC/myeloid/conventional), monocyte related (MoDC, CD14^+^) and tissue resident (based on anatomical location; Kratochvilova and Slama, 2018). Cattle DC generated from bone marrow cultures can be characterized as CD1^high^, MHC class II^high^, CD80^high^, CD86^high^, CD11a^high^, CD11b^intermediate^, CD11c^low^, and CD14^low^ (De Carvalho et al., 2006). DC derived from monocytes express myeloid-associated markers (CD11a, CD11b, CD14 and CD172a) and upregulate CD40, CD80, and CD86 upon activation. Tissue distribution of bovine DC can also be assessed phenotypically. Peripheral blood DC can be characterized by expression of MHC class II, CD11c, and CD172a; DC in the thymic medulla express CD1 and CD172a; and DC in Peyer's patches express CD11b and CD172a (Bimczok et al., 2005).

In peripheral blood, two main subgroups of bovine DC have been characterized: plasmacytoid DC (pDC) and conventional DC (cDC). Bovine pDC were defined as CD4^+^, CD80^med^, DEC205^+^, and similar to human and mice, produce large amounts of type I interferons in response to TLR-agonists (Sei et al., 2014; Reid et al., 2016). Bovine cDC express MHC class II but can be further subdivided into two subgroups. One subgroup is phenotypically characterized as CD11c^+^ MHC class II^+^ CD80^+^, DEC205^+^, CD172a^+/−^, and CD16^+/−^. Upon TLR-stimulation, this subgroup upregulates CD80 expression and produces large amounts of TNF-α. This subset is thought to be specialized in antigen presentation to naïve T cells (Sei et al., 2014). Additionally, CD11c^+^ cDC are highly efficient at antigen internalization. The second subgroup represents a population of CD11c^−^ MHC class II^+^ DEC205^+^ DC that appear to be the precursors for CD11c^+^ cDC (Sei et al., 2014). In other species, strategies to target nanovaccines to particular DC subsets have shown promising results (Wattendorf et al., 2008; Hamdy et al., 2011). In cattle DC, similar strategies are likely to be useful for nanovaccine development as their ability to preferentially uptake nanoparticles was successfully investigated *in vitro* using a DEC205 mAb (Walters et al., 2015).

### The Bovine Adaptive Immune System

#### Cellular Immunity

Cattle have two main subsets of CD3^+^ T cells: alpha beta (αβ) and gamma delta (γδ) T cells. Like other species, αβ T cell subsets in cattle include CD4^+^ helper T cells and CD8^+^ cytotoxic T cells. CD4^+^ T cells and CD8^+^ T cells are MHC restricted in cattle and recognize antigenic peptides that are presented in the context of MHC class I and MHC class II. CD4^+^ T cells play a role in the activation of other immune cells such as CD8^+^ T cells and macrophages via secretion of soluble cytokines. CD4^+^ T cells are also critical for B cell differentiation and production of high-affinity isotype switched antibodies. Cytotoxic CD8^+^ T cells have an important role in killing virally infected cells.

Memory CD4^+^ and CD8^+^ T cell responses are important to sustain the protection induced by vaccines. In cattle, as in humans and rodents, two functionally distinct subsets of memory CD4^+^ T cells have been described (Maggioli et al., 2015): T central memory (Tcm) cells and T effector memory (Tem) cells. Tcm cells have a high proliferative capacity and long life span, and are therefore associated with long-term protection; while Tem cells are less proliferative, but demonstrate more immediate effector functions (Sallusto et al., 2010). Tcm cells in cattle express CD45RO, CCR7, and high levels of CD62L, while Tem cells express CD45RO, low to intermediate levels of CD62L and are CCR7 negative (Maggioli et al., 2016). CD4^+^ Tcm cells have been correlated with protection against *M. bovis* (Maggioli et al., 2016) and *Mycoplasma mycoides* (Totte et al., 2013), the causative agent of contagious bovine pleuropneumonia. Memory CD8^+^ T cells have also been described in cattle, although the characteristic Tcm and Tem subsets are less well-defined (Hogg et al., 2009; Svitek et al., 2018). Another memory population thought to be important for long-term protection, particularly in the context of respiratory infections, are tissue-resident memory CD4 and CD8 T cells. While these populations have been defined in mice and humans (Snyder and Farber, 2019), they have not yet been elucidated in cattle.

One important aspect of the bovine cellular immune system which differs from that of rodents and humans is the predominance of γδ T cells. In ruminants, γδ T cells constitute a major lymphocyte population in peripheral blood, epithelial tissues, and sites of inflammation (Hein and Mackay, 1991), comprising up to 40–50% of all circulating lymphocytes in young calves. The predominance of γδ T cells in the bovine suggests that they play an important role in host defense, particularly in young animals. γδ T cells possess multiple effector functions, including cytokine production, cytotoxicity, and immune regulation (Guerra-Maupome et al., 2019b). Bovine γδ T cells also have the capacity for immune memory, mounting enhanced secondary responses to infections months to years after vaccination or a primary infection (Blumerman et al., 2007; Guerra-Maupome et al., 2019a). γδ T cells in ruminants have the capacity to recognize protein and non-protein ligands [reviewed in Born et al. (2013)], including soluble and lipid fractions from many pathogens, although ruminant γδ T cells but do not appear to recognize phosphoantigens, which is the primary antigen recognized by human γδ T cells (Vesosky et al., 2004). Bovine γδ T cells respond to pathogen associated molecules via a variety of PRR, including TLR2, 3, 4, and 7, and NOD1 and NOD2 (Hedges et al., 2005; McGill et al., 2013). Bovine γδ T cells also express the workshop cluster 1 (WC1) receptor, a member of the scavenger-receptor cysteine rich superfamily. WC1 acts as both a co-receptor and pattern recognition receptor on bovine γδ T cells (Hsu et al., 2015). Their capacity to integrate signals via both the T cell receptor and their PRR makes γδ T cells an attractive target for vaccine development in cattle. To date, however, there are few targeted attempts to engage the bovine γδ T cell population in vaccine-mediated protection.

#### Humoral Immunity

##### Ultralong CDR H3 Bovine Antibodies

Cattle have the same classes of antibodies (Ab) that have been described in the most studied mammalian species, humans and mice, namely IgM, IgA, IgD, IgG, and IgE. However, diversification of the antibody repertoire due to V(D)J recombination in cattle is not as robust as it is in these species as a result of fewer VH segments, as well as conservation among those sequences of the segments that are involved in the rearrangement process (Zhao et al., 2006). Studies have demonstrated that somatic hypermutation and junctional nucleotide additions and deletions are primary diversifiers of the bovine Ab repertoire (Kaushik et al., 2009; Liljavirta et al., 2014). Interestingly, bovine Ab contains long CDR H3s, with substantial diversity generated in these regions through somatic hypermutation (Kaushik et al., 2009). There exists within the bovine CDR H3 multiple hotspots with a codon bias toward cysteine mutations (Stanfield et al., 2018). In humans, IgVH CDR3 is typically 8–16 aa in length, whereas, in cattle lengths exceeding 50 aa are quite common, including a repertoire subset that contains ultralong CDR H3 of >70aa. The ultralong CDR H3 has an unusual architectural structure that extends out from the surface of the antibody molecule comprised of a β-strand “stalk” and a “knob” domain, which is connected via multiple disulfide bonds. The stalk and knob in the ultralong CDR H3 Ab evidently mediate antigen binding, as suggested by the sequencing of multiple clones derived from BVDV-vaccinated cattle (Wang et al., 2013). These authors have theorized that cattle Ab with ultralong CDR3 might be more effective at neutralizing certain viruses, such as BVDV, by binding to conserved epitopes not easily accessible on the viral surface by conventional Ab. Adding further uniqueness to the bovine Ab repertoire is the more recent discovery of two functional bovine IgM genes, located in tandem in a single IgH locus (Ma et al., 2016), the only such existence found among mammalian species. It is interesting to note that ultralong CDR H3 Ab appear to predominantly utilize IgM2 rather than IgM1 and there is greater length variation in the CDR H3 of IgM2 (Stanfield et al., 2018). The resulting structural variability would significantly increase the potential for diversity in the bovine CDR H3 region, of importance in antigen binding. To date, there has been no systematic examination of ultralong CDR H3 Ab in response to nanovaccine immunization and such an investigation is warranted, especially for vaccines targeting viral pathogens.

##### Bovine Maternally Derived Antibodies

Like other species, the immune system of the newborn calf is immature and inherently skewed toward a fetal T helper 2 (Th2) immunity with minimal Th1 responses, rendering them more susceptible to respiratory and diarrheal infections (Chase et al., 2008; Chattha et al., 2009, 2010; Siegrist and Aspinall, 2009). Maternally-derived antibodies (MDA) protect the neonate from infections during this infancy period but decline over time, presenting an opportunity for infection (Krakowka et al., 1978; Nyiro et al., 2017). In cattle, colostral transfer of antibodies is the major source of passive immunity, and these Ab are predominately of the IgG1 subclass, with minor amounts of other antibodies, which differs from several other mammalian species. While their presence is critical to the health of the newborn, MDA have also been reported to hamper vaccination-induced responses, even when present at very low titers. Many examples exist that demonstrate the inhibitory effects of MDA in humoral and cell-mediated immunity in veterinary vaccines following parenteral vaccination such as Foot and mouth disease virus (Patil et al., 2014), BVDV (Endsley et al., 2003; Downey et al., 2013), and BRSV (Ellis et al., 2010, 2013; van der Sluijs et al., 2010). These studies report that the presence of high titers of maternal antibodies at the time of immunization correlates with inhibition of seroconversion after vaccination and suppression of vaccine responses. A reliable vaccine for young stock should overcome the challenge of MDA interference. Mucosal delivery of vaccines is more likely to “override” the presence of passively acquired MDA to induce a protective immune response in young animals, as reported in several studies such as BVDV (Blodörn et al., 2014) and BRSV (Vangeel et al., 2007; Ellis et al., 2013; McGill et al., 2018, 2019).

### The Bovine Mucosal Immune System

The bovine mucosal immune system is characterized by mucosa-associated lymphoid tissues (MALTs) very rich in T cells, B-cells, plasma cells, and APCs as in other species. They are located in strategic sites in the hosts' oral, gut, genitourinary, and respiratory tracts and are interconnected for efficient antigen sampling [reviewed in Chase and Kaushik, 2019].

A major goal of mucosal immunization is to induce high titers of neutralizing antibodies at barrier surfaces, thus blocking further pathogen invasion. Previously, it was reported that IgG1 is the major Ig in nasal and tracheal secretions in calves through 6 months of age (Morgan et al., 1981). As newborn calves essentially lack plasma cells in the nasal passages (Morgan et al., 1981), the source of IgG1 found in these tissues has been debated (Ellis et al., 2018). A recent study clearly demonstrated the re-secretion of passive IgG1 in the nasal mucosa of neonatal calves (Ellis et al., 2018). In a review, it was noted that in the calf's upper respiratory tract, secretory IgA is the primary antibody (Osman et al., 2018), which suggests that IgA increases with the age of the calf. Certainly, in adult cattle, it is known that similar to other mammalian species, IgA predominates in the upper respiratory tract. In the palatine and pharyngeal tonsils of the calf, cytoplasmic IgG^+^ cells were found, with few IgA^+^ cells, in the light zone of germinal centers by 3 months of age (Yasuda et al., 2006). In addition, these authors further detected IgG mRNA in the germinal centers and parafollicular areas of both tonsils and IgA mRNA in the parafollicular area of the pharyngeal tonsil. On the other hand, it has been established that IgG1 is the predominant antibody in the lower respiratory tract of cattle, due to both local production and systemically derived Ig (Morgan et al., 1981). IgG1 responses in sera and colostrum from cows and IgA in nasal secretions and saliva from their calves against intimin were induced following intranasal vaccination of both groups with recombinant *Escherichia coli* intimin adjuvanted with a modified heat labile enterotoxin (Yokomizo et al., 2002). A strong induction of local IgA responses was observed following colonization of the bovine terminal rectal mucosa by *E. coli* O157/H7, with responses directed against multiple Ag, including type III secretion-dependent proteins, LPS, flagellin and outer membrane proteins (Nart et al., 2008). Evidently, depending on the age, tissue sampled, and secretions examined, IgG1 and IgA can play critical roles in bovine mucosal immunity. Therefore, both classes of Ab should be examined in studies of vaccines administered via mucosal routes.

### Mucosal Vaccination Strategies

Many pathogens infect the host via mucosal sites that act as barriers between the host and the external environment. Traditionally, many licensed vaccines are administered parenterally by intramuscular or subcutaneous injections, which are effective in inducing a systemic protective immune response but may fail to elicit much-needed antigen-specific responses at the mucosal entry sites. In contrast, mucosal vaccination can induce detectable local immune responses in tissues and systemic responses in the blood without being invasive [reviewed in Rhee (2020)]. To induce mucosal immunity, the vaccine is delivered in a particulate form with an adjuvant via a suitable device at the target site where the antigens are taken up by APCs. Unlike parenteral vaccination with injectable liquids, mucosal vaccination formulations may include, pressurized nasal sprays, nebulizers, or dry powder formulations. These allow for improved vaccine stability, especially thermal stability, if the antigens are either stabilized and dried or liquids lyophilized to enhance storage stability (Hellfritzsch and Scherließ, 2019). Previous reviews by Partidos and Hickey also highlight the advantages of mucosal vaccination from a regulation and production standpoint such as less cost of mass immunization, which is an attractive trait for livestock farmers; and non-invasiveness which eliminates a potential source for infections, and vaccine-site reactions which may adversely impact meat quality (Partidos, 2000; Lu and Hickey, 2007). Although tremendous progress has been made in mucosal immunology in the last decade, only a few successful commercial mucosal vaccines are available for use in the veterinary field [reviewed in Wilson et al. (2020)]. Nanovaccines are particularly well-suited to mucosal vaccine strategies, further underlining their potential usefulness for vaccine development in cattle and other production animals.

## Vaccine Safety and Vaccine Enhanced Disease

One significant consideration for the design of novel vaccine platforms is the potential for vaccine-enhanced disease. Though not widely seen, there have been several reports of vaccine-enhanced disease in cattle, either observed in experimental studies with bacterins (Wilkie et al., 1980) or killed virus vaccines (Gershwin et al., 1998; Kalina et al., 2004) or noted, for example, in field cases following BRSV infection (Kimman et al., 1989; Schreiber et al., 2000). Some early research with *Mannheimia haemolytica* bacterins administered through differing routes showed these vaccines to enhance protection against virulent challenge (Confer et al., 1987; Purdy et al., 1997). However, there were also situations in which calves were not protected or in which enhanced disease was observed. For instance, calves receiving a formalin-inactivated *M. haemolytica* vaccine subcutaneously had more severe clinical disease following challenge compared to non-vaccinated controls (Wilkie et al., 1980). An early account of severe respiratory disease in the field that was likely enhanced by vaccination with BRSV was reported by researchers in the Netherlands (Kimman et al., 1989). Calves vaccinated with a modified live BRSV 2 days prior to the occurrence of the outbreak had more severe clinical signs than those observed in non-vaccinated calves, suggesting that the modified live vaccine administered during the course of natural infection may have enhanced the disease. Similarly, Schreiber et al., reported higher mortality (30%) in calves during a BRSV outbreak that had received an inactivated BRSV vaccine 3–4 months earlier than that found in calves not vaccinated (0%). An interesting finding was significant infiltration of eosinophils in the lungs of the vaccinated calves (Schreiber et al., 2000). Furthermore, a vaccine-enhanced disease associated with BRSV has been replicated under experimental conditions (Gershwin et al., 1998; Kalina et al., 2004), with evidence of a bias toward a Th2 response. This would be in fitting with the increased eosinophilia observed by Schreiber et al. (2000). In contrast, a separate investigation found an early enhancement of clinical signs of respiratory disease in response to BRSV following use of a formalin-inactivated vaccine, but vaccinated calves ultimately had reduced lung pathology compared to controls (West et al., 1999). Though not frequently encountered, this highlights the importance of understanding the pathogens' immunopathology mechanisms and the safety of potential vaccine candidates.

## Why Nanovaccines?

Although traditional vaccine formulations composed of attenuated or inactivated virus are effective at promoting long-lasting immunity, safety concerns persist. Attenuated vaccine formulations require strict storage conditions to maintain vaccine potency. Violation of adherence to demanding storage protocols can cause the vaccine to become contaminated with the bioactive form of the pathogen (Vartak and Sucheck, 2016). Further, attenuated formulations are not applicable for immunosuppressed and fatal pathogens such as human immunodeficiency virus (HIV), Hepatitis C, or *Mycobacterium tuberculosis*. Subunit vaccines address the challenges previously mentioned but are limited in immunogenicity and ability to stimulate long-term cellular and humoral immunity (Vartak and Sucheck, 2016). Further, soluble proteins used in subunit vaccine formulations typically have short half-lives *in vivo* and are often poorly immunogenic, requiring multiple booster shots to elicit long-lasting immunity (Wilson-Welder et al., 2009; Vartak and Sucheck, 2016). Within recent decades, numerous studies have been dedicated to the design of use of novel subunit vaccine delivery systems to overcome these shortcomings.

The self-adjuvanting effects, release kinetics, and delivery routes of nanoparticles can be manipulated by particle size, shape, surface charge, and hydrophobicity (Kumari et al., 2010). Size determines the overall biodistribution and internalization of nanovaccines by immune cells. Particles in the 20–100 nm size range have the ability to directly enter the lymphatic system within a few hours of administration while 200–500 nm particles must be internalized by antigen presenting cells to reach the lymphatic system (Bachmann and Jennings, 2010). Generally, particles on the micron scale are successfully internalized by antigen presenting cells while larger particles (>10 micrometers) show difficulty in internalization (Thomas et al., 2011; Ramirez et al., 2017). Recent studies focused on the effect of particle shape found greater rates of internalization by dendritic cells with rod-shaped particles compared to spherical particles (Sharma et al., 2010; Jindal, 2017). Further studies have also shown the ability of rod-shaped particles to circulate within the gastrointestinal tract and blood for longer periods of time when compared to spherical particles (Zhao Y. et al., 2017). When considering surface charge, both anionic and cationic particles are internalized by antigen presenting cells, although tracking studies have shown cationic particles escaping from lysosomes (Thomas et al., 2011; Yue et al., 2011; Carrillo-Conde et al., 2012; Ramirez et al., 2017). Particles synthesized from hydrophobic polymers are more readily phagocytosed when compared to hydrophilic formulations (Thomas et al., 2011; Shima et al., 2013). Hydrophobicity also controls the antigen release kinetics from particles with hydrophobic polymers decreasing antigen release rates (Lopac et al., 2009; Huntimer et al., 2013a; Haughney et al., 2014).

The choice of polymer plays a large role on the immunogenic effects caused by the particles, but particle synthesis also allows for great variety in terms of the choice of encapsulated or loaded particulate materials. Particles can be formulated to encapsulate or have a surface coating composed of proteins, adjuvants, or both. Particles are further capable of simultaneously loading multiple proteins (Wu et al., 2014). In the context of animal vaccines, the use of marker vaccines, also known as “differentiating infected from vaccinated animal” (DIVA) vaccines, is desirable for their ability to distinguish between infected and vaccinated animals (Lidder and Sonnino, 2012). DIVA vaccines are formulated with multiple proteins and contain at least one protein less antigenic than the wild-type virus (Lidder and Sonnino, 2012). Polymeric particles are capable of simultaneous loading with various proteins without sacrificing loading efficiency of each individual protein, making them an attractive candidate for DIVA vaccines (Wu et al., 2014; Ross et al., 2016; Zacharias et al., 2018). We next describe some polymer chemistries that have been used to formulate vaccines used in cattle as listed in Table 1.

**Table 1:** Nanoparticles/nanocarriers chemistries used for formulating vaccines for different cattle diseases.

**Polymer type**	**Polymer**	**Structure**	**Characteristics**	**References**
Polyester (Synthetic)	PLGA	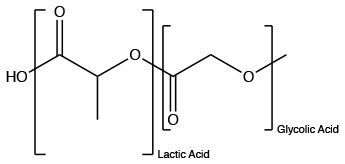	Allows for control of hydrophobicity, stimulates IgG	Makadia and Siegel, 2011
Polyanhydrides (Synthetic)	CPTEG	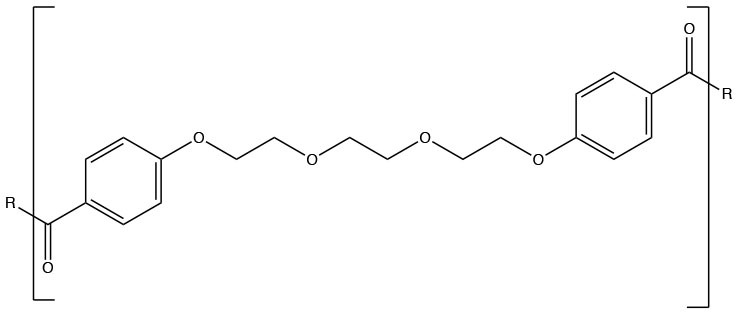	Hydrophilic and relatively quick degradation profile	Carrillo-Conde et al., 2010
	CPH	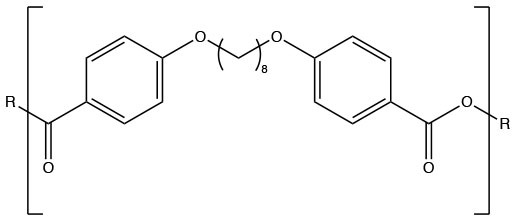	Hydrophobic will a relatively slow degradation profile	Carrillo-Conde et al., 2010
	SA	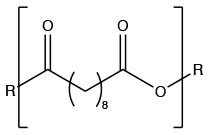		Carrillo-Conde et al., 2010
Polysaccharide (Natural)	Chitosan	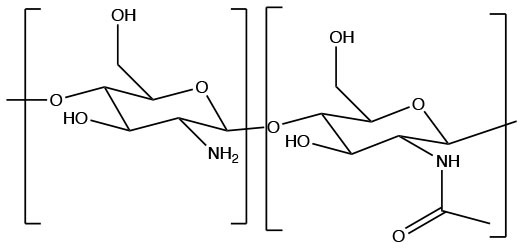	Cationic, biodegradable, induces humoral and cellular immune response, insoluble at physiological pH	Islam and Ferro, 2016

## Polymeric Nanovaccine Chemistries

The following sections will focus on polymeric particles due to their ability to provide sustained release of antigenic payloads, stabilize such payloads, and induce enhanced antibody- and cell-mediated immune responses, both systemically and locally. Polymeric particles allow for modulation of various characteristics, such as size, shape, hydrophobicity, and surface charge. Manipulation of these characteristics influences the immunogenicity of the particles. Particle size, shape, and surface charge affect their cellular internalization (Thomas et al., 2011; Carrillo-Conde et al., 2012; He and Park, 2016; Ramirez et al., 2017). Sub-micron particles are readily internalized by antigen presenting cells to induce a robust immune response, while particles >10 μm are not internalized by antigen presenting cells (Thomas et al., 2011; Ramirez et al., 2017). Rod shaped particles are readily internalized by antigen presenting cells, including dendritic cells and macrophages, while spherical particles show decreased rates of internalization in comparison (Gratton et al., 2008; Sharma et al., 2010; Banerjee et al., 2016; He and Park, 2016; Jindal, 2017). Both anionic and cationic particle induce significant immune responses (Thomas et al., 2011; Carrillo-Conde et al., 2012). Tracking studies have shown the ability of cationic particles to escape lysosomes while neutral and anionic particles localize within the lysosomes (Yue et al., 2011). Particle hydrophobicity can be directly tailored by variations in polymer chemistry. Hydrophobic particles with relatively high molecular weights undergo increased rates of cellular uptake compared to their hydrophilic counterparts (Thomas et al., 2011; Shima et al., 2013; Goodman et al., 2014).

Polymeric particles encapsulating antigen can be synthesized by a variety of methods, including emulsion-based methods, salting out, nanoprecipitation, and spray drying (Determan et al., 2006; Lopac et al., 2009; Sosnik and Seremeta, 2015). The more common benchtop methods, which are based on single and double emulsions, have demonstrated success in producing particles with varying payloads, but have limitations with respect to their scalability. Scaling up emulsion methods could lead to batch variability between particles and high manufacturing and solvent costs (Thao Truong-Dinh et al., 2016; Martínez Rivas et al., 2017). Spray drying is a relatively new but promising method to producing antigen encapsulated polymer particles. Spray drying produces particles by atomization of suspended antigen and polymer. Spray drying holds multiple advantages over traditional benchtop methods, including its potential for scalability, low energy, low cost, and no requirement for further product purification (Sosnik and Seremeta, 2015; Arpagaus et al., 2018). With a variety of synthesis methods, particle surfaces can also be functionalized with ligands for specific targeting to C-type lectin receptors (CLRs), Toll-like receptors (TLRs), or Nod-like receptors (NLRs). Multiple functionalization techniques are available based on the size and type of ligand. Polymeric particles have been functionalized with dimannose to target CLRs and TLRs (Tamayo et al., 2010; Carrillo-Conde et al., 2011; Chavez-Santoscoy et al., 2012; Vela Ramirez et al., 2014; Phanse et al., 2017; Rintelmann et al., 2019). These particles successfully activated DCs and CD8+ T cells while up-regulating MHC II and pro-inflammatory cytokines (Tamayo et al., 2010; Carrillo-Conde et al., 2011; Vela Ramirez et al., 2014; Phanse et al., 2017). Further studies have targeted TLRs by functionalizing particles with CpG nucleotides to elicit a Th1 immune response (Demento et al., 2010). NLR targeting ligands have been co-encapsulated into polymeric particles with antigen to elevate antibody response (Pavot et al., 2013).

Antigen delivery by polymeric particles for bovine diseases is a relatively new field of study with limited, but promising results. A comprehensive summary of immune response to varying particle formulations in bovine and other animal models can be found in Table 2. Polymeric particles delivered intranasally to calves induced significant amounts of nasal IgA (Kavanagh et al., 2003, 2013; Mansoor et al., 2014; Pan et al., 2014; McGill et al., 2018, 2019). Similarly, intramuscular delivery of particles induced both mucosal and systemic antibody responses (Riffault et al., 2010, 2020). Subcutaneous delivery of a polyanhydride vaccine implant to bovine species induced CD4+ T cell production and a humoral response (Boggiatto et al., 2019). These studies showed the potential of utilizing polymeric particles against bovine disease.

**Table 2:** List of antigens/payloads delivered by using different nanocarriers/particles for the treatment of different cattle diseases.

**Material**	**Size (nm)**	**Pathogen**	**Animal model**	**Adjuvant**	**Antigens/payloads**	**Route of administration**	**Immune responses induced in the host**	**References**
Silica nanoparticles	40–50	BVDV	Sheep Mice	NP as the carrier and adjuvant NP as the carrier and adjuvant	E2 membrane structural glycoprotein	Subcutaneous Subcutaneous	Th1 and Th 2 mediated responses Antibody and T-cell mediated responses	Mahony et al., 2014, 2015; Zhang et al., 2014
		*Anaplasma marginale*	Mice	NP as the carrier and adjuvant	VirB9-1 and/or VirB9-2 Outer membrane proteins	Subcutaneous	Both B-cell and T-cell responses	Zhao et al., 2016; Zhao L. et al., 2017
Poly (lactic-co-glycolic acid), PLGA	225.4	BPI3V	Bovine Mice Bovine	NP as the carrier and adjuvant	OVA BPI3V proteins Heat-inactivated, purified whole BPI3V virus	Intranasal	Induced Ovalbumin-specific IgA in nasal secretions and serum IgG responses. Induced local immune responses, IgA. Induced Ag-specific IgA response in the mucosa	Kavanagh et al., 2003; Mansoor et al., 2014, 2015
		*Brucella spp*	Mice	NP as the carrier and adjuvant	Brucella oligopolysaccharide (OPS) Ribosomal proteins rL7/L12	Intraperitoneal	Induce humoral responses Induce both humoral and cellular responses/Th1 responses	Singh et al., 2015; Maleki et al., 2019
		BRSV	Bovine	NP as the carrier and adjuvant	Post fusion (F) protein and attachment (G) protein	Intranasal	Induce local immune responses, IgA No infection challenge in this study	Kavanagh et al., 2013
Chitosan-coated PLGA		FMDV	Bovine	NP as the carrier and adjuvant	Protein, P1-2A, and the viral protease 3C protein	Intranasal	Induced strong nasal IgA response and cellular immune responses	Pan et al., 2014
Polyanhydride	200–800	BRSV	Bovine	NP as the carrier and adjuvant	Post fusion (F) protein and attachment (G) protein	Intranasal	Induce both local and systemic (IgA and IgG) humoral immune responses and cellular responses	McGill et al., 2018, 2019
		*Mycobacterium avium* subsp. *paratuberculosis*	Mice	NP as the carrier and adjuvant	Whole cell lysate and culture filtrate	Subcutaneous	Induction of polyfunctional T cell responses	Thukral et al., 2020
Polyanhydride CPTEG:CPH polymer-Vaccine Platform for Extended Antigen Release (VPEAR)		*Brucella spp*	Bovine	NP as the carrier and adjuvant	Methanol-killed RB51 antigens	Subcutaneous	Induce CD4+ T cell and humoral responses	Boggiatto et al., 2019
N-nanorings	15	BRSV	Bovine Bovine Mice	Montanide^TM^ ISA 61 VG Montanide™ ISA71 VG 5% Montanide™ Gel 01	BRSV- pre-F and N subunit BRSV-F and -G epitopes FsII neutralizing epitope from antigenic site II of the post F protein	Intramuscular (IM) Intramuscular (IM) or Intranasal Intranasal	Induced stronger mucosal BRSV and preF IgA responses Stimulated mucosal and systemic Ab responses and cellular immunity Induced antigen specific humoral responses	Riffault et al., 2010, 2020; Hervé et al., 2017
Calcium phosphate nanoparticles (CaPNs)		*B. abortus*	Mice	NP as the carrier and adjuvant	*Brucella* antigens (FliC,7a-HSDH, BhuA) and multi-epitopes (Poly B and poly T)	Intraperitoneal	Induced increase in cellular and humoral immune responses. Balanced Th 1 (IgG2a) and Th 2 (IgG1) mediated responses was also observed.	Sadeghi et al., 2020b
Chitosan nanoparticles		*B. abortus*	Mice	NP as the carrier and adjuvant NP as the carrier and adjuvant	Flagellin FliC Malate dehydrogenase (Mdh)	Subcutaneous Intranasal	Induced humoral and cellular immune responses/ Th1 immunity. Induced Th2-related immune responses and systemic IgA (respiratory tract, digestive and genital mucosa)	Soh et al., 2019; Sadeghi et al., 2020a
Poly (diaminosulfide) (PNSN) microparticles-VPEAR		*L. borgpetersenii* serovar Hardjo strain HB15B203 (L203)	Bovine	NP as the carrier and adjuvant Seppic Montanide, Alhydrogel	Whole lysate Whole lysate	Subcutaneous Subcutaneous	Induced humoral responses and adaptive T-cell- and B-cell-mediated immune responses Induce both humoral and cellular responses	Wafa et al., 2020; Wilson-Welder et al., 2020

### Synthetic Polymers

#### Polyesters

Poly(lactic-co-glycolic acid) (PLGA) is a promising biodegradable and biocompatible polyester (Danhier et al., 2012; Silva et al., 2016). PLGA is composed of the biodegradable monomers, lactic acid and glycolic acid (Allahyari and Mohit, 2016). Generally, these particles undergo bulk erosion to maintain steady release of encapsulated antigen (Allahyari and Mohit, 2016). The release rate of PLGA particles can be directly manipulated by varying the ratio of lactic acid to glycolic acid (Allahyari and Mohit, 2016). Hydrophilic particles with higher amounts of glycolic acid show a larger burst and surface release of antigen (Thomas et al., 2011; Allahyari and Mohit, 2016). Numerous studies have demonstrated the immunological benefits of utilizing PLGA particles to combat varying bovine bacterial and viral diseases, such as foot-and-mouth disease (FMD), diarrhea virus, and parainfluenza (Fowler et al., 2012; Pan et al., 2014; Mansoor et al., 2015; Walters et al., 2015).

#### Poly(diamosulfides)

Poly(diaminosulfide) (PNSN) is a less studied biodegradable and biocompatible polymer originally synthesized for the purpose of drug delivery (Yoo et al., 2012; D'Mello et al., 2014). This polymer remains stable when exposed to organic solvents, degrades quickly within acidic environments, and maintains low toxicity levels *in vivo* (Yoo et al., 2012; D'Mello et al., 2014). The backbone composition of this polymer (i.e., N and S) allows for rapid degradation in acidic conditions. This may serve as an advantage for internalized particles exposed to acidic environments within phagosomal locations inside dendritic cells, stimulating the quick release of antigen (D'Mello et al., 2014). Recent studies have shown robust bovine immune responses to leptospirosis antigen encapsulated PNSN microparticles (Wafa et al., 2020; Wilson-Welder et al., 2020).

#### Polyanhydrides

Polyanhydrides are biocompatible and biodegradable polymers that have been used for both drug and vaccine delivery applications. Particles synthesized from polyanhydrides are advantageous in terms of antigen stability, sustained antigen release, induction of both antibody and cell mediated immunity, and enhanced immunity dependent on route of administration (Petersen et al., 2010, 2012; Huntimer et al., 2013b; Ross et al., 2014; Vela Ramirez et al., 2014; McGill et al., 2018; Liu et al., 2020). As a unique feature, polyanhydride nanovaccines maintain antigen stability following room temperature storage, making them ideal for storage at locations not capable of maintaining a cold chain (Wagner-Muñiz et al., 2018). Polyanhydride particles degrade through surface erosion into non-toxic and easily metabolized by-products (Lopac et al., 2009). Particles synthesized from polyanhydrides allow for the direct manipulation of hydrophobicity and antigen release rates by varying the ratio of copolymers. These copolymers are generally composed of 1,8-bis(*p*-carboxyphenoxy)-3,6-dioxaoctane (CPTEG), 1,6-bis(*p*-carboxyphenoxy) hexane (CPH), and sebacic anhydride (SA). Of the three copolymers, CPH is the most hydrophobic and particles rich in CPH content degrade very slowly (~months) while particles rich in CPTEG and SA are the more hydrophilic and result in the fastest degradation profiles (~days to weeks) (Lopac et al., 2009). Manipulation of release rates and hydrophobicity has a direct effect on the immune response elicited, with the hydrophobic formulations eliciting robust long-lasting humoral and cellular immunity, potentially obviating the need for booster vaccinations (Ulery et al., 2011; Wafa et al., 2017).

Multiple studies have analyzed the promising use of polyanhydride particles to combat bovine diseases, such has respiratory syncytial virus and mycobacterium subspecies paratuberculosis (McGill et al., 2018, 2019; Thukral et al., 2020). The use of a CPTEG:CPH-based polyanhydride implant has also shown enhanced immunity against Brucella spp (Boggiatto et al., 2019; Wilson-Welder et al., 2020).

### Natural Polymers

#### Chitosan

Chitosan is a natural biopolymer extensively used in the agricultural, biomedical, and pharmaceutical fields with recent studies analyzing its use for drug delivery (Aranaz et al., 2009; Elgadir et al., 2015). Chitosan is a cationic biodegradable, biocompatible, and non-toxic polymer (Bernkop-Schnürch and Dünnhaupt, 2012; Ahmed and Aljaeid, 2016). These characteristics allow for controlled drug release and mucoadhesion (Bernkop-Schnürch and Dünnhaupt, 2012).

## Examples of Nanovaccines That Have Been Explored in Cattle

Several nanovaccine platforms and candidate nanovaccines have been tested for their capacity to mitigate infectious diseases in cattle. Table 2 depicts a summary of available published reports, the nanoparticle platform that was implemented and the disease that was targeted.

### Viral Diseases

Viral diseases are a leading cause of significant economic losses in the cattle industry worldwide, and prevention of these viral diseases using vaccines is essential. Some of the clinically relevant viruses in cattle include Bovine Viral Diarrhea Virus (BVDV-1), Bovine Respiratory Syncytial Virus (BRSV), Bovine ParaInfluenza 3 (BPI3V), Bovine Adenovirus, and Bovine Herpesvirus-1 (BHV-1), all of which may play a role in the bovine respiratory disease complex (BRDC). Primary respiratory viral infection predisposes the animal to secondary bacterial infections which causes severe morbidity, mortality, and production losses. While multiple live and inactivated vaccines are available for use against each of these viruses, numerous reports indicate variable efficacy for these traditional vaccines under field conditions. Compounding this problem, the cost of multiple boosts adds a considerable financial burden to the producer. Several recent reports have investigated the efficacy of a nanovaccine approach for the prevention of BRDC-associated viral infection.

Bovine Viral Diarrhea Virus (BVDV-1) is a virus that causes gastrointestinal, respiratory, and reproductive infections in all ages of cattle and sheep, including unborn calves through the placenta (Brock, 2003). The E2 membrane structural glycoprotein has been identified as a highly immunogenic glycoprotein that elicits neutralizing antibodies (Bruschke et al., 1997; Thomas et al., 2009; Pecora et al., 2012; Snider et al., 2014) which protect against experimental BVDV-1 challenge (Snider et al., 2014). Mesoporous silica nanoparticles (MSNs) have been tested as an adjuvant and antigen carrier for the BVDV-1 E2 glycoprotein. Mahony and colleagues demonstrated that *in vivo* delivery of a recombinant BVDV-E2 nanovaccine using hollow MSNs in mice elicited cellular virus specific IFN-γ secretion by T cells and humoral total IgG, immune responses (Mahony et al., 2014), with cellular immunity sustained for at least 6 months after immunization (Mody et al., 2015). In a follow-up study using sheep, the authors reported that immunization with the BVDV-E2 MSN nanovaccine induced no adverse effects and stimulated of both arms of the immune system, with cell mediated immune responses being sustained for at least 4 months after immunization (Mahony et al., 2015). In this study, they also demonstrated that the freeze-dried BVDV-E2 MSN nanovaccine protected the integrity of the recombinant protein and was stable at room temperature for as long as 14 months (Mahony et al., 2015).

PLGA-based biomimetic nanoparticles have also shown promise for use against BVDV in a calf model. Biomimetic particles are formulated to encapsulate multiple antigens and a defined adjuvant in a spatial arrangement that is designed to mimic the pathogen itself. Riitho and colleagues designed a biomimetic PLGA particle that encapsulated the BVDV-1 NS3 protein, a known T cell target of the virus, and poly(I:C) as the adjuvant, and then coated with the structural envelope E2 glycoprotein (Riitho et al., 2017). A subcutaneous prime-boost with the biomimetic particle induced strong neutralizing antibody responses and E2 and NS3-specific T cell responses in calves. The protection afforded by the experimental nanovaccine was similar to that induced by a commercial, inactivated BVDV vaccine (Riitho et al., 2017).

Bovine Respiratory Syncytial Virus (BRSV) causes severe acute lower respiratory tract disease in young calves and contributes to BRDC in cattle of all ages. Vaccine development for BRSV must be particularly targeted to young animals due to this increased susceptibility. Thus, an efficient BRSV vaccine must be effective even in the presence of maternal antibodies. McGill et al. recently developed a 50:50 CPTEG:CPH-based polyanhydride nanovaccine that encapsulated the recombinant post-fusion F and G proteins from BRSV (BRSV-F/G). The nanoparticle encapsulation maintained the immunogenicity of the post-fusion F and G antigens and resulted in sustained release kinetics of the antigens over a 30-days *in vitro* period (McGill et al., 2018). A single, intranasal immunization of the BRSV-F/G nanovaccine in 3-weeks old, colostrum-replete calves induced virus-specific cellular responses in the peripheral blood and lower respiratory tract (LRT) together with virus-specific IgA responses in both nasal secretions and bronchoalveolar lavage fluid. These immune responses correlated with reduced virus burden, shedding, and lung pathology in vaccinated calves compared to non-vaccinated calves following a BRSV challenge 4 weeks later (McGill et al., 2018, 2019). Kavanagh et al. demonstrated that intranasal delivery of two synthetic peptides from the BRSV F and G proteins in PLGA nanoparticles induces mucosal IgA responses, but no detectable serum IgG or cellular immune responses. The animals in this study were not experimentally challenged, so it is unknown if the IgA response was sufficient to induce some degree of protection against BRSV infection (Kavanagh et al., 2013).

The internal N nucleoprotein of BRSV is a major target for CD8^+^ cytolytic responses in calves (Samal et al., 1991), hence a potential vaccine candidate. Due to the tendencies of RSV-N protein to form nucleocapsid-like structure, a nanoparticle was created by Tran et al. by fusing the C-terminal of the human RSV P phosphoprotein with the RSV N protein co-expressed in *E. coli* resulting in recombinant soluble N protein assembled in 15 nm diameter ring-like structures that can be used as a potential vaccine candidate (Tran et al., 2007). In cattle, vaccination with the N-nanorings provides partial protection against experimental BRSV challenge and induces strong cell mediated immune responses (Riffault et al., 2010). The RSV-nanorings can also be used as carriers for other immunogenic peptides. They have been engineered to carry epitopes from the BRSV F and G proteins (Blodörn et al., 2014), and induced partial protection against BRSV challenge in calves. In mice, N-nanorings carrying the FsII neutralizing epitope from antigenic site II of the F protein, the same epitope recognized by the monoclonal antibody Palivizumab (SYNAGIS®, AstraZeneca), induced partial protection against HRSV infection (Hervé et al., 2017). More recently, the N-nanorings were co-administered with the prefusion F protein from BRSV and shown to induce complete protection from the experimental challenge (Riffault et al., 2020).

Bovine Parainfluenza 3 Virus (BPI3V) constitutes another persistent health challenge to the cattle industry. It causes infections in the upper respiratory tract (URT) of 2- to 8-months-old beef and dairy cattle (Caswell and Williams, 2016). Similar to the instance with BRSV and other common respiratory viruses, the presence of circulating maternal antibodies interferes with an efficient immune response to the vaccine (Fulton et al., 2004; Morein et al., 2007). However, the use of nanoparticles as vaccines carriers delivered directly to the mucosal surface is expected to overcome this hurdle and can target the antigen to specific immune cells (Wattendorf et al., 2008; Hamdy et al., 2011). An early study using intranasal immunization with a PLGA nanoparticle encapsulating OVA reported induction of the desired mucosal immune response in calves, characterized with ovalbumin-specific IgA in nasal secretions and serum IgG responses (Kavanagh et al., 2003). In mice, intranasal delivery of the immunodominant hemagglutinin-neuraminidase (HN) and fusion (F) glycoproteins encapsulated in PLGA nanoparticles induced a stronger and more rapid serum IgG response compared to soluble antigen alone (Mansoor et al., 2014). In a follow-up study, the same authors compared the immune responses induced by the commercial, intranasal live attenuated BPI3V vaccine (Rispoval RS + PI3 intranasal, Zoetis), to those against a PLGA nanoparticle vaccine encapsulating heat-inactivated, purified whole BPI3V virus in dairy calves (Mansoor et al., 2015). Calves receiving the PLGA nanovaccine demonstrated a more sustained antigen-specific IgA response in the mucosa even in the presence of pre-existing virus-specific mucosal IgA and systemic IgG, a good trait for a candidate vaccine (Mansoor et al., 2015). However, in the future, the efficacy of the BPIV3 PLGA nanovaccine should be assessed.

Foot and Mouth Disease Virus (FMDV) is a highly infectious virus that causes fever and blister-like sores on the mouth and hooves of the animal. FMDV infection causes high mortality in young animals and is linked to significant production losses in adult animals. FMDV can be transmitted via exposure to contaminated aerosols, and studies have shown that neutralizing antibody responses in the mucosa are sufficient to prevent infection. Pan *et al*. generated two separate nanoparticle platforms for mucosal immunization against type A FMDV (Pan et al., 2014). The first was a chitosan-coated PLGA particle loaded with plasmid DNA encoding the capsid precursor protein, P1-2A, and the viral protease 3C protein from the virus. The second was a chitosan-trehalose particle encapsulating whole, inactivated FMDV. The authors used a natural exposure model by co-housing the immunized animals with two infected animals. The PLGA nanoparticles induced the strongest nasal IgA response and most potent cellular immune responses, while both nanoparticle formulations induced low serum IgG responses. Neither group of vaccinated animals was fully protected against viral shedding or clinical disease; however, the animals receiving the PLGA particles were significantly improved over the control animals and those cattle receiving the chitosan-trehalose nanoparticle formulations (Pan et al., 2014).

### Bacterial Diseases

Infectious diseases have an economic impact through animal loss, decreased animal productivity, and treatment costs. In addition, many bacterial diseases of cattle are zoonotic and therefore have the potential to spread to humans and further the economic burden due to human morbidity, mortality, and cost of therapy. In many cases, it is known that the best measure to control human disease is to control disease in animal populations (Olsen and Stoffregen, 2005), yet successful vaccine strategies have not yet been developed for many of these bacterial diseases. Below are some examples of bacterial diseases of cattle and recent nanovaccine approaches for disease control.

Johne's disease is a highly contagious, chronic infection caused by *Mycobacterium avium* subspecies *paratuberculosis*. The bacteria target the small intestine of ruminants causing chronic enteritis, diarrhea, weight loss, decreased production, and eventually death in affected animals. There are no treatments for Johne's disease, and disease control is mediated through the culling of infected animals. Polyanhydride nanoparticles have been tested as a potential vaccine strategy for Johne's in the mouse model. Thukral et al. utilized 20:80 CPTEG:CPH nanoparticles containing *M. paratuberculosis* cell lysate and culture filtrate proteins as a vaccine in mice (Thukral et al., 2020). The vaccine was found to be well-tolerated (i.e., no inflammatory reactions at the site of administration) and induced polyfunctional CD8^+^ T cells characterized by the production of IFN-γ, IL-2, and TNF-α. Additionally, following challenge with *M. paratuberculosis*, the authors observed a reduction in bacterial loads in multiple organs, suggesting some level of protection (Thukral et al., 2020).

Brucellosis is a disease caused by infection with bacteria of the genus *Brucella*. In domestic animals, including cattle, *Brucella* infection primarily results in reduced milk production, infertility, and reproductive losses either as late term abortions, stillbirth, or weak calves. As with other intracellular pathogens, protection from infection requires the induction of T helper 1 (T_H_1) responses, characterized by IFN-γ producing CD4^+^ T cells and cytotoxic CD8^+^ T cells. Various nanovaccine formulations against brucellosis have been reported in the mouse model. PLGA particles carrying *Brucella* oligopolysaccharide (OPS) (Maleki et al., 2019) or *Brucella* ribosomal proteins rL7/L12 (Singh et al., 2015) have been tested in mice and were shown to induce humoral and cellular responses. Protection against a virulent challenge, demonstrated by reduced bacterial loads in the spleen of challenged mice was also shown (Singh et al., 2015). Similar findings were also shown using calcium phosphate nanoparticles (CaPNs) adsorbed with three *Brucella* antigens (FliC, 7a-HSDH, BhuA) and two multi-epitopes (poly B and poly T) (Sadeghi et al., 2020b) and with mannosylated chitosan nanoparticles carrying FliC (Sadeghi et al., 2020a). Mucoadhesive chitosan nanoparticles loaded with *B. abortus* malate dehydrogenase (Mdh) have been tested in their ability to induce mucosal and systemic immune responses following intranasal administration in mice (Soh et al., 2019).

Despite the extensive use of nanoparticle platforms for vaccination against brucellosis in mice, their potential for generating protective immune responses in cattle have not yet been explored. Recently, however, Boggiatto et al. utilized a formulation of 20:80 CPTEG:CPH nanoparticles as a vaccine platform for extended antigen release (VPEAR) using killed *Brucella abortus* strain RB51 antigen in cattle (Boggiatto et al., 2019). This single dose administration delivers antigen in three ways: (1) soluble killed RB51 antigen administered subcutaneously, (2) a depot of killed *Brucella abortus* strain RB51 antigen in 20:80 CPTEG:CPH rod to serve as a boost, and (3) a slow-release depot of RB51 antigen in 20:80 CPTEG:CPH within a polyethylene capsule. In this study, the authors were able to demonstrate that memory CD4^+^ T cell and IgG responses were elicited with a killed vaccine approach following challenge of animals with live RB51 vaccination. These responses were long-lived (i.e., measured 18 months post-vaccination), and while protection from challenge with a virulent field strain of *Brucella* was not tested, this was the first time that a killed vaccine succeeded in engaging cellular immune responses in cattle. While not nanoscale, these data highlight the potential applicability of biodegradable polymers as vaccine platforms.

Leptospirosis is a zoonotic disease caused by infection with spirochete bacteria of the genus *Leptospira*, which includes more than 250 pathogenic serovars (Evangelista and Coburn, 2010; Lehmann et al., 2014). In dairy and beef herds, *L. borgpetersenii* serovar Hardjo is the most common cause of infection. During infection, leptospiral organisms colonize the genital tracts and the kidney's proximal tubules, allowing them to be shed in genital and urinary tract secretions. In cattle, infection with *Leptospira* can result in reduced milk production and reproductive failure. In addition, cattle can shed bacteria and be asymptomatic chronic hosts, serving as a source of infection for other animals and for humans (Yupiana et al., 2019). Conserved leptospiral antigen, shared by multiple serovars have been identified, but tend to be poorly immunogenic, and would therefore benefit from a delivery platform that could enhance those responses. Vaccination of cattle using *L. borgpetersenii* serovar Hardjo strain HB15B203 (L203) antigen derived from whole cell sonicate encapsulated in poly(diaminosulfide) (PNSN) microparticles, was shown to elicit *Leptospira*-specific immune responses characterized by CD3^+^ and CD21^+^ cells, and increased levels of agglutinating IgG1 and IgG2 anti-L203 antibodies (Wafa et al., 2020). In a different study also using L203 antigen encapsulated in PNSN, CD4^+^ and γδ T cells proliferative responses, as well as IgG1 and IgG2 responses (Wilson-Welder et al., 2020) were measured. While these vaccination strategies successfully engaged cell-mediated and humoral immunity, information regarding homologous and heterologous protection as well as length of immunity remains to be determined.

Wilson-Welder et al. also tested the VPEAR platform to deliver killed L203 antigens (Wilson-Welder et al., 2020). The authors demonstrated that following *in vivo* antigen challenge with a *Leptospira* Hardjo bacterin (Spirovac, Zoetis), CD4^+^, CD8^+^, and γδ T cells responses were present in VPEAR-vaccinated animals. Production of IgG1, and to a lesser extent, IgG2 could also be measured in VPEAR-vaccinated animals following antigenic challenge (Wilson-Welder et al., 2020). Altogether, the data would suggest that this alternative polyanhydride-based platform elicits cellular and humoral responses to killed leptospiral antigen.

Bovine anaplasmosis, or “tick fever,” is a tick-transmitted disease with worldwide distribution and poses a major burden to beef producers with an estimated annual loss of $300 million in the United States alone (Uilenberg, 1995; Kocan et al., 2003). The disease is caused by infection with the rickettsial hemoparasite *Anaplasma marginale*, which invades red blood cells and causes severe anemia. Currently, there are no safe and efficacious vaccines and control measures involve the administration of low doses of in-feed chlortetracycline for several months (Reinbold et al., 2010), which as a result of the veterinary feed directive (VFD), requires a veterinary oversight and therefore creates an increased burden on producers. Outer membrane (OM) proteins of *A. marginale* have been shown to induce homologous protection against infection, characterized by antigen specific CD4^+^ T cells and IgG2 production in calves (Brown et al., 1998). However, purification of OM proteins is cost prohibiting, therefore, the use of subunit vaccines in combination with novel delivery platforms is particularly promising. Nano platforms have been explored in *A. marginale* vaccines including silica vesicles (SV100) carrying type IV secretion system proteins (T4SS) VirB9-1 and VirB9-2 (Zhao et al., 2016; Zhao L. et al., 2017), and VirB9-1 and VirB10 proteins (Zhang et al., 2016), as well as carbon nanotubes carrying the major surface protein (MSP)1a (Pimentel et al., 2020), in the mouse model. These formulations induce both cell-mediated and humoral responses that are protective against challenge; however, they have not been tried in cattle.

Curtis et al. developed a subcutaneous implant utilizing 20:80 CPTEG:CPH copolymer to deliver MSP1a in cattle (Curtis et al., 2020). As described above, this VPEAR strategy is a single dose vaccine that delivers soluble antigen, provides an antigenic boost, and serves as an antigen depot for long-term release. In this study, the authors showed the ability of VPEAR to provide long-lived protection (i.e., 21 months) from challenge with *A. marginale*, demonstrated by diminished clinical signs. However, in this study, unlike previous reports with brucellosis (Boggiatto et al., 2019) and leptospirosis (Wilson-Welder et al., 2020), some animals rejected the VPEAR implant. Overall, while further studies are needed to increase the immunogenicity of anaplasmosis subunit vaccines, these new vaccination strategies appear very promising.

## Conclusions and Future Prospects

Compared to traditional vaccines the use of the nanoparticle platform as vaccines carriers and /or adjuvants have significant potential to improve vaccines by providing stability outside the cold chain, enhancing immunogenicity of their payload, engaging both humoral and cellular immune responses, and promoting long-lived responses without the need for booster doses. As evidence from the literature, there is significant interest in various adaptive nanoparticle platforms to use in cattle and other production animals. There are a number of considerations for the future if nanovaccines are to become mainstream in the veterinary field. Of utmost importance is the feasibility of industrial scale-up. Traditional bench-top emulsion methods used to prepare nanovaccines pose several concerns for scale-up, including high costs and batch-to-batch variability. Further, scaling up would require significantly large volumes of solvent, lengthy drying times, and large containers to accommodate solvent volumes. Scale-up could quickly become exhaustive from a materials and processing standpoint (Martínez Rivas et al., 2017). However, recently developed spray-drying processes are low cost, low energy and readily scalable (Sosnik and Seremeta, 2015; Arpagaus et al., 2018). Spray-drying approaches also minimize the need for further product purification and ensure more uniform batches. Materials such as solvent and polymers are readily available and relatively inexpensive. Thus, although large-scale manufacturing of nanovaccines is not currently practiced, it is an attainable goal.

To date, nearly all efforts aimed at nanovaccine development in cattle have focused on a univalent formulation, targeted to a single pathogen. Currently on the market, vaccines for diseases such as BRDC, which involve multiple pathogens, are multivalent products that are designed to protect against as many as 5–8 individual pathogens. This approach minimizes handling, the number of vaccines that must be administered, and ensures the animal has been immunized against several possible pathogens prior to exposure. If a nanovaccine formulation is to be successful in today's market, it will be essential to explore the efficacy and immunogenicity of multivalent nanovaccine formulations for preventing disease complexes. One opportunity for enhancing the performance of various nanovaccine platforms is the use of cell-targeting approaches. In murine studies, there has been extensive investigation into the use of cell-targeting techniques such as monoclonal antibodies or functionalized carbohydrate decorations that ensure optimal delivery of the vaccine to the desired immune cell. In cattle, one *in vitro* study has revealed that an antibody against DEC205 can successfully target the vaccine for more efficient uptake by DC (Walters et al., 2015); however, this idea has yet to be applied to *in vivo* immunogenicity studies in cattle. Cell-targeting techniques are expected to be particularly useful when considering mucosal vaccination strategies, to ensure targeted delivery of aerosol, intranasal or oral vaccines.

Another area of future promise is the possibility of nanoparticle-based platforms for drug delivery vehicles. Promising studies from rodents have shown that nanoparticles can be highly effective drug-delivery vehicles that can target drugs to intracellular compartments, thus increasing bioavailability and reducing toxicity associated with systemic treatment. In cattle, there is a significant effort aimed at reducing the use of antimicrobials to reduce the risk of antimicrobial resistance and reduce drug residues in meat and milk. Use of a targeted, drug-delivery nanoparticle system could be exploited to deliver therapeutics directly to the site of infection or targeted to a particular organism, thus reducing systemic drug residues and minimizing the dose of drug necessary for effect. In cases such as mastitis, which can be difficult to treat with antibiotics, targeted delivery of a controlled release, drug-delivery nanoparticle could be more effective. There is also growing interest in the field of immunomodulatory drugs in the field of cattle health. As with drug delivery, use of a nanoparticle delivery system could be used to deliver immunomodulatory drugs to targeted organs or sites of infection, and provide sustained release over a period of time while the animal may be at high-risk for disease or infection.

In summary, nanovaccines offer several benefits over traditional adjuvants. There have been a number of studies demonstrating that nanovaccines have the potential for enhanced immunogenicity and efficacy in cattle. Further studies investigating the use of nanovaccines for disease prevention or therapies in production animals such as cattle are warranted.

## Author Contributions

TM, EG, PB, RS, BN, and JM conceptualized and outlined the manuscript. TM, PB, RS, and JM wrote the sections focused on bovine immunology and examples of nano vaccines in cattle. EG and BN wrote the sections focused on the advantages of nanovaccines and nanovaccine formulations. All authors edited the manuscript.

## Conflict of Interest

The authors declare that the research was conducted in the absence of any commercial or financial relationships that could be construed as a potential conflict of interest.
